# Supplementary material for: Excited-State Dynamics of All the Mono-cis and the Major Di-cis Isomers of β-Apo-8′-Carotenal as Revealed by Femtosecond Time-Resolved Transient Absorption Spectroscopy
Source: Molecules. 2023 May 29;28(11):4424. doi: 10.3390/molecules28114424 (PMC10254598; doi:10.3390/molecules28114424)
Supplement: Supplementary file 1 [file molecules-28-04424-s001.zip › molecules-2381469-supplementary.pdf]

Supplementary Materials

1. The way how to determine the peak wavelengths of the vibrational structures of the steady-state absorption spectra of the isomers of β-apo-8'-carotenal.

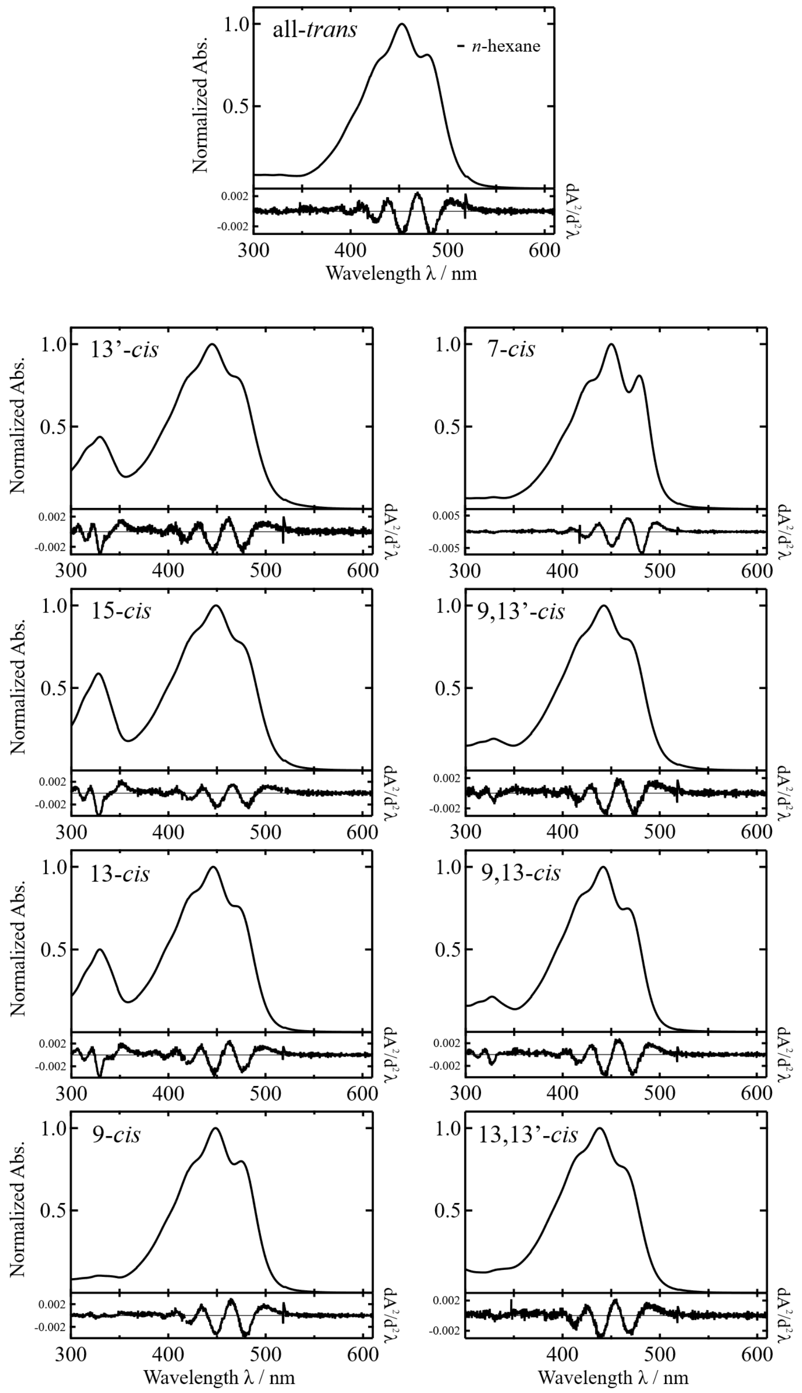

(a)

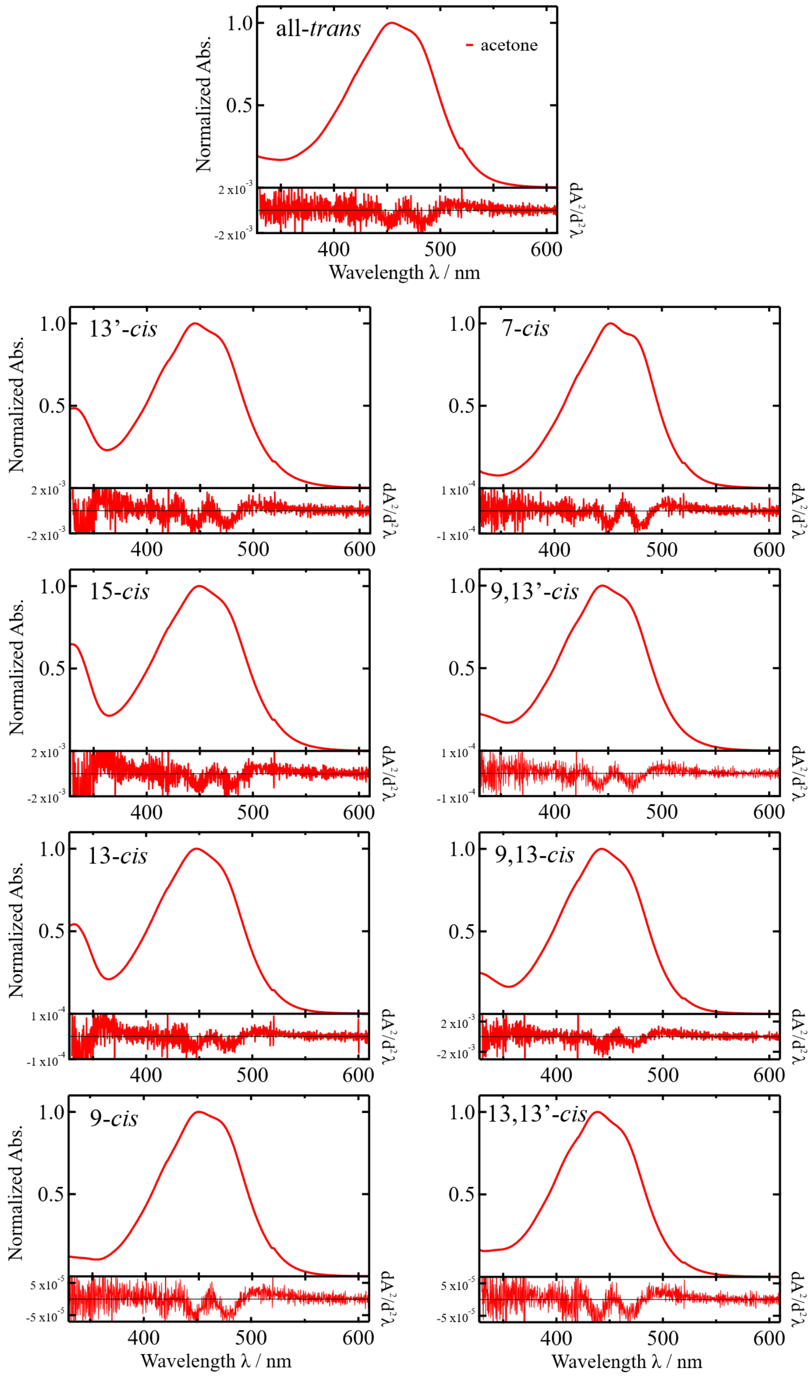

all-trans
acetone
13'-cis
7-cis
15-cis
9,13'-cis
13-cis
9,13-cis
9-cis
13,13'-cis
Normalized Abs.
Wavelength λ / nm

(**b**)

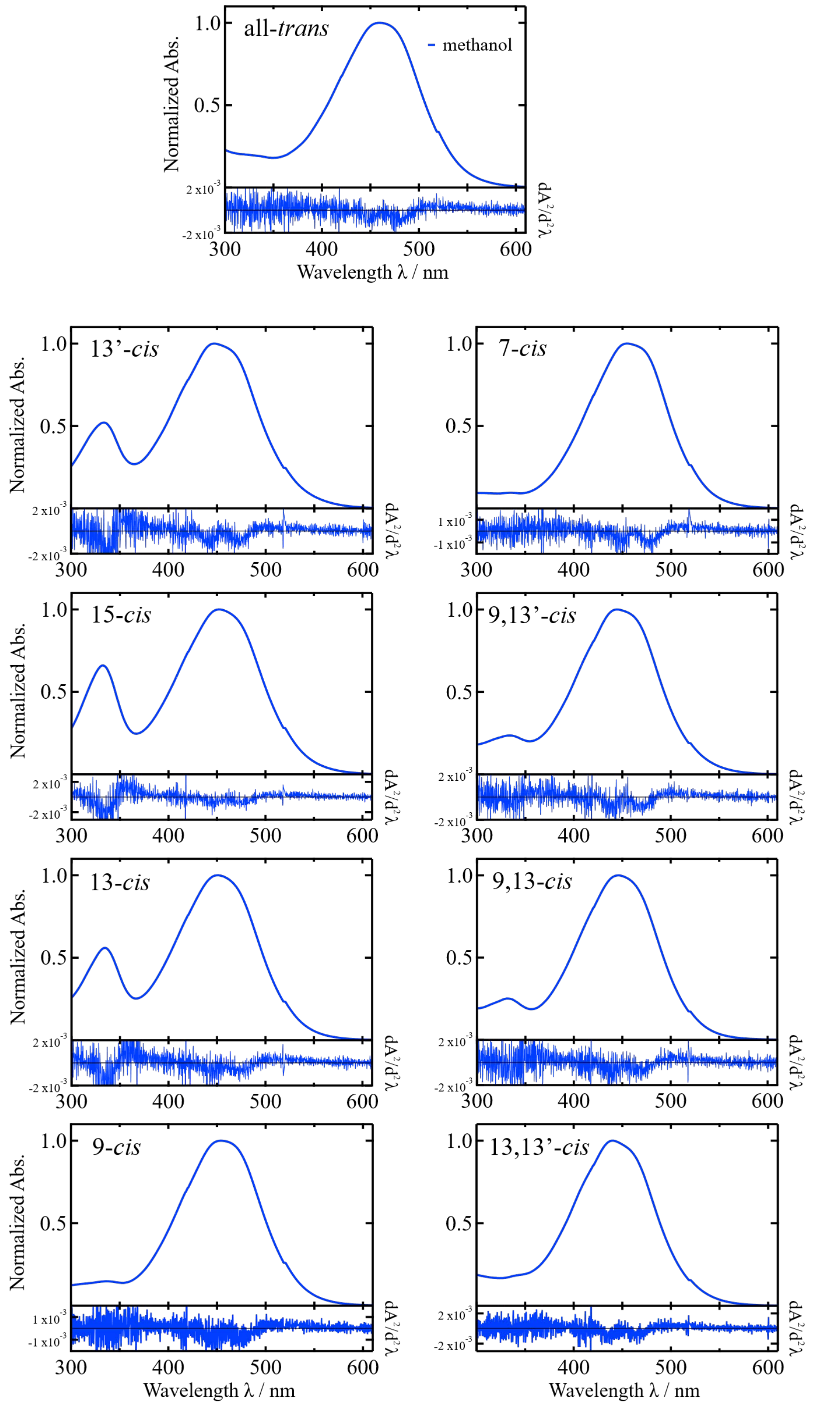

(c)

Figure S1. (a). The steady-state absorption spectra of the all-*trans*, 13′-*cis*, 15-*cis*, 13-*cis*, 9-*cis*, 7-*cis*, 9,13′-*cis*, 9,13-*cis*, and 13,13′-*cis* isomers of β-apo-8′-carotenal in *n*-hexane (solid black-line) recorded at room temperature. The second derivative waveforms of the absorption spectra ($dA^2/d^2\lambda$) are shown at the bottom of each panel. **(b).** The steady-state absorption spectra of the all-*trans*, 13′-*cis*, 15-*cis*, 13-*cis*, 9-*cis*, 7-*cis*, 9,13′-*cis*, 9,13-*cis*, and 13,13′-*cis* isomers of β-apo-8′-carotenal in acetone (solid red-line)

recorded at room temperature. The second derivative waveforms of the absorption spectra ($dA^2/d^2\lambda$) are shown at the bottom of each panel. **(c).** The steady-state absorption spectra of the all-*trans*, 13′-*cis*, 15-*cis*, 13-*cis*, 9-*cis*, 7-*cis*, 9,13′-*cis*, 9,13-*cis*, and 13,13′-*cis* isomers of β-apo-8′-carotenal in methanol (solid blue-line) recorded at room temperature. The second derivative waveforms of the absorption spectra ($dA^2/d^2\lambda$) are shown at the bottom of each panel.

Figure S1-a shows the steady-state absorption spectra of the isomers of β-apo-8'-carotenal in *n*-hexane. The peak wavelengths of the vibrational structures in absorption spectra were determined by calculating the second derivative waveforms the absorption spectra. The minima observed in the second derivative waveforms correspond to the peaks of the vibrational structures. Figure S1-b and Figure S1-c show the similar protocols to determine the peak wavelengths of the vibrational structures in absorption spectra in acetone and methanol.

2. Comparison of the intensity ratios of the *cis*-band/man-band transitions against C6-C8' distances of the isomers of β-apo-8'-carotenal in *n*-hexane, acetone, and methanol.

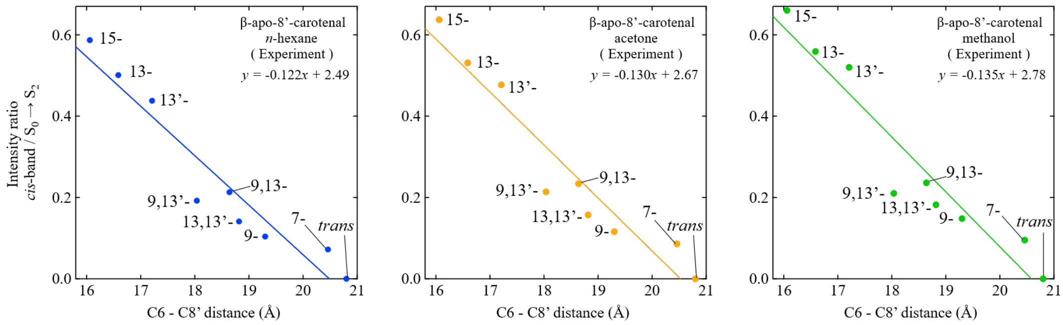

Figure S2. Relationships between the C6-C8' distance of the geometrically optimized structures of the isomers β-apo-8'-carotenal and the ratio of experimentally determined intensity of the *cis*-band transition divided by that of the $S_0 \rightarrow S_2$ transition of the isomers in *n*-hexane, acetone, and methanol solutions at room temperature.

As illustrated in Figure S2, the intensity ratios of the *cis*-band/main band transitions against the C6-C8' distances show good linear relationships for the isomers of β-apo-8'-carotenal in *n*-hexane, acetone, and methanol. It is interesting to note that the magnitudes of the slopes similar values among different solvents, which suggests that the ratio of the oscillator strengths of *cis*-band transitions to those of the main-band transitions might be maintained in different solvents with different polarities.

3. Experimentally observed femtosecond time-resolved absorption spectra of the isomers of β-apo-8'-carotenal.

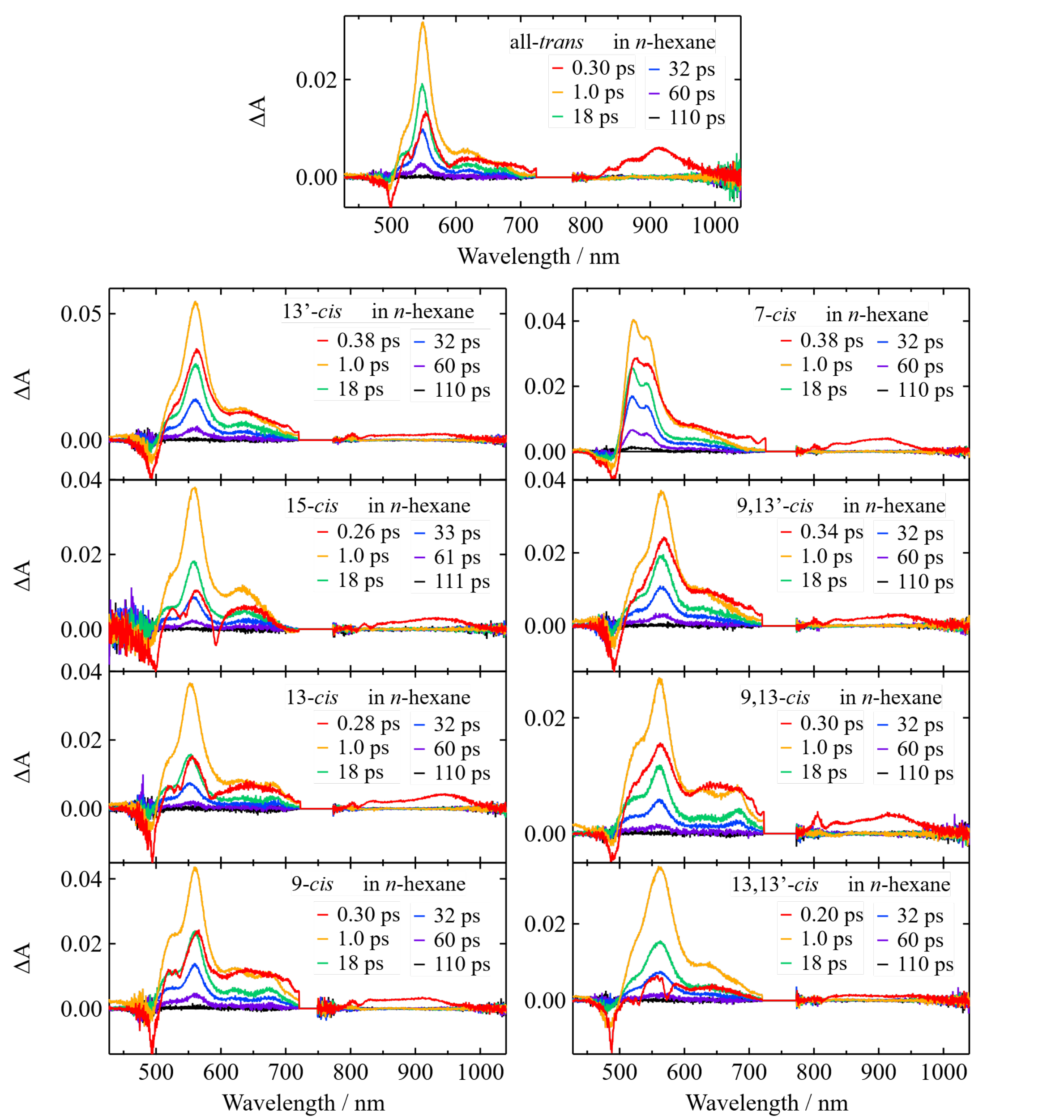

(a)

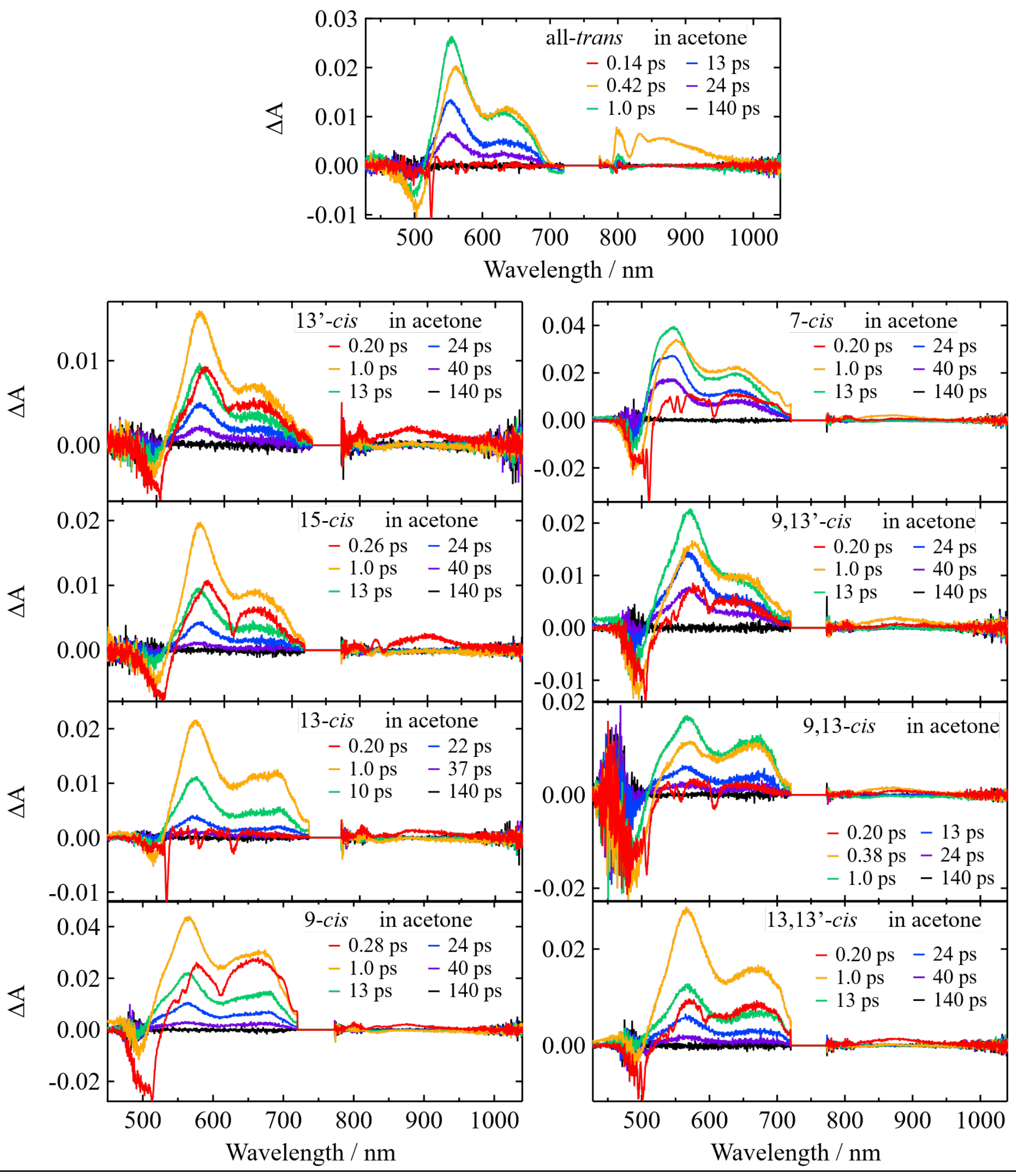

all-*trans* in acetone
0.14 ps
0.42 ps
1.0 ps
13 ps
24 ps
140 ps
ΔA
Wavelength / nm
13'-*cis* in acetone
0.20 ps
1.0 ps
13 ps
24 ps
40 ps
140 ps
7-*cis* in acetone
15-*cis* in acetone
0.26 ps
9,13'-*cis* in acetone
13-*cis* in acetone
22 ps
37 ps
10 ps
9,13-*cis* in acetone
0.38 ps
9-*cis* in acetone
0.28 ps
13,13'-*cis* in acetone

(b)

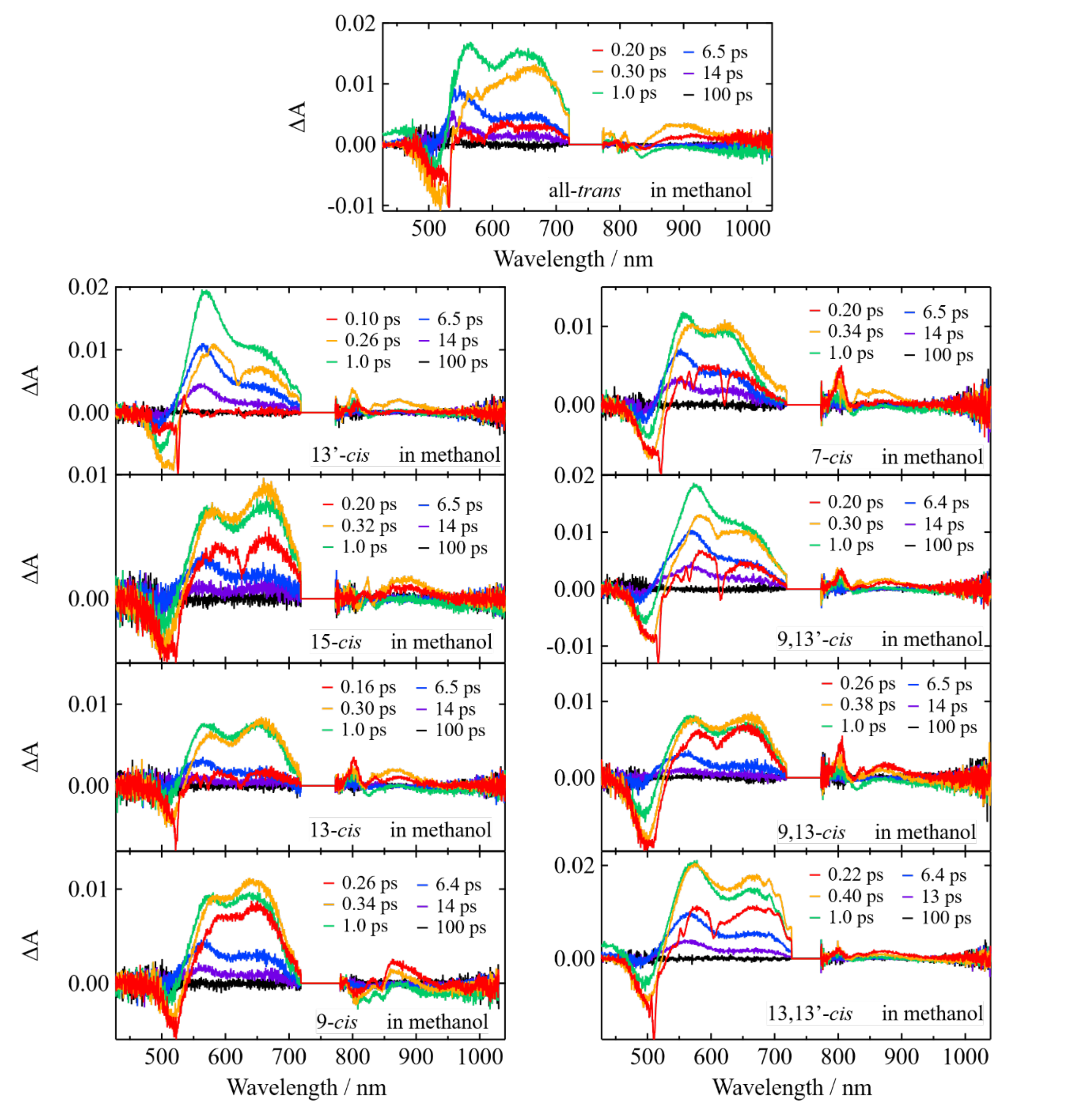

(c)

Figure S3. (a). Femtosecond time-resolved absorption spectra of the all-*trans*, 13′-*cis*, 15-*cis*, 13-*cis*, 9-*cis*, 7-*cis*, 9,13′-*cis*, 9,13-*cis*, and 13,13′-*cis* isomers of β-apo-8′-carotenal in *n*-hexane recorded at selective delay times at room temperature. **(b).** Femtosecond time-resolved absorption spectra of the all-*trans*, 13′-*cis*, 15-*cis*, 13-*cis*, 9-*cis*, 7-*cis*, 9,13′-*cis*, 9,13-*cis*, and 13,13′-*cis* isomers of β-apo-8′-carotenal in acetone recorded at selective delay times at room temperature. **(c).** Femtosecond time-resolved absorption spectra of the all-*trans*, 13′-*cis*, 15-*cis*, 13-*cis*, 9-*cis*, 7-*cis*, 9,13′-*cis*, 9,13-*cis*, and 13,13′-*cis* isomers of β-apo-8′-carotenal in methanol recorded at selective delay times at room temperature.

4. Evolution associated difference spectra (EADS) derived by applying the global analysis with three components sequential model to the entire datasets of the femtosecond time-resolved absorption spectra of the isomers of β-apo-8′-carotenal.

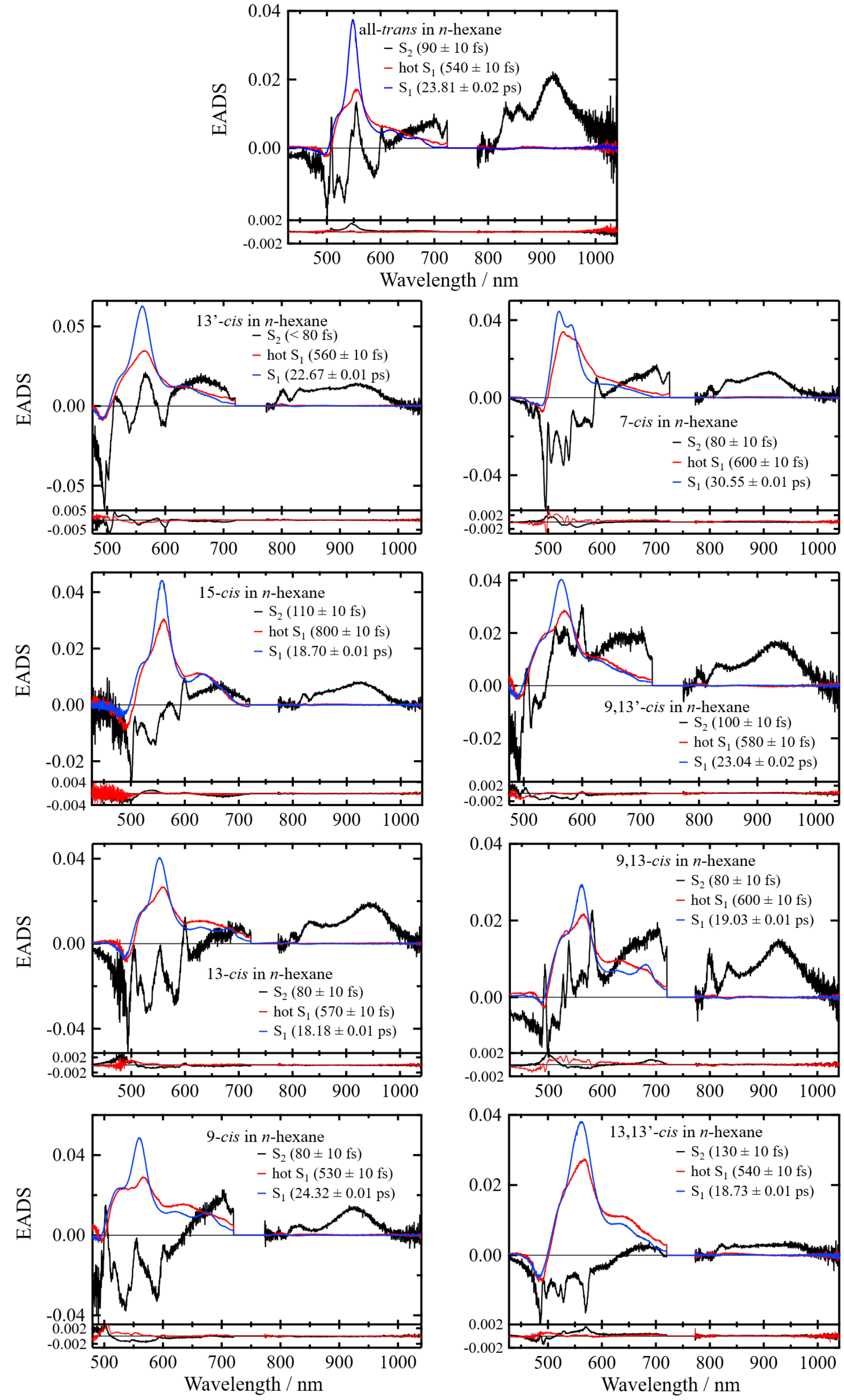

(a)

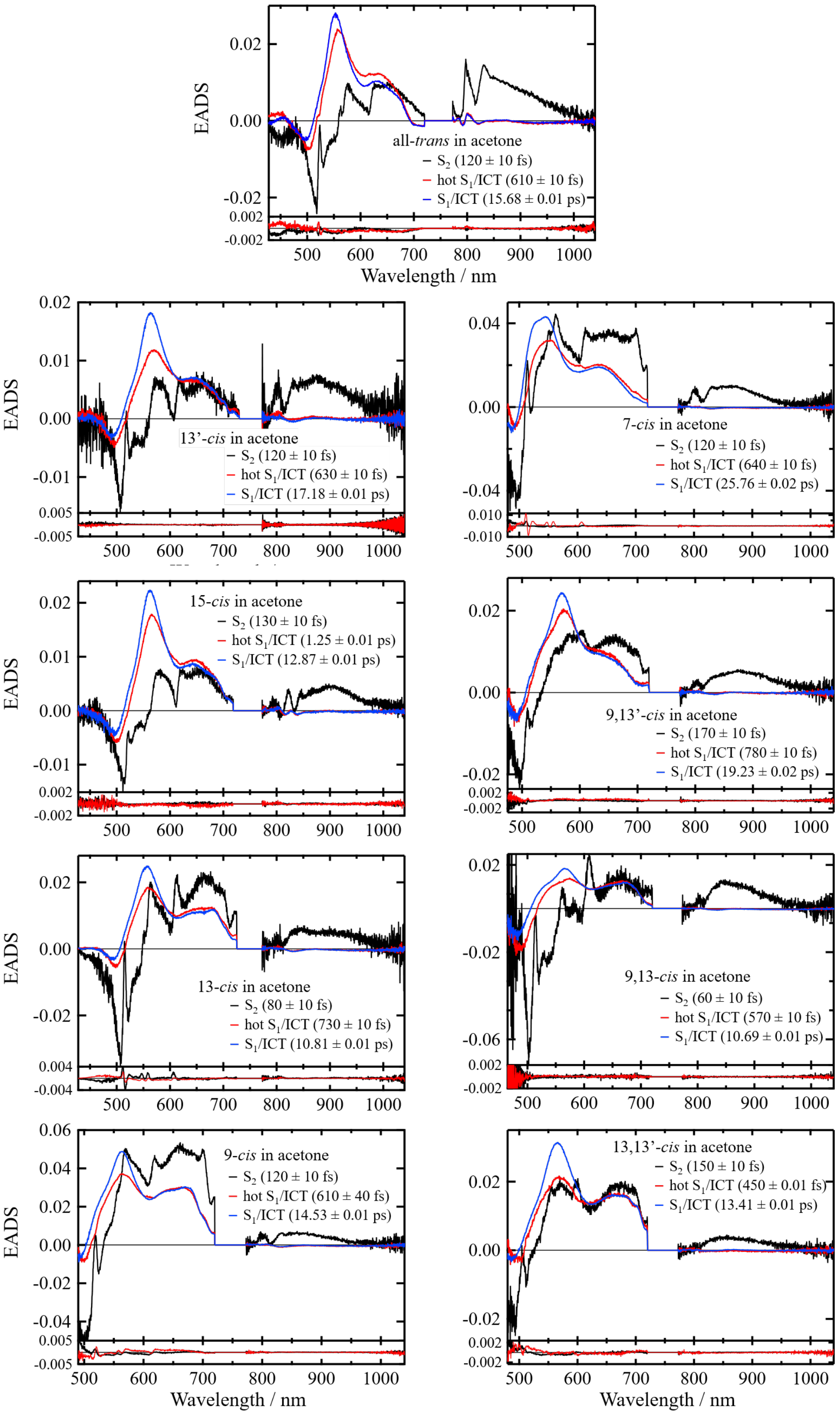
EADS
Wavelength / nm
all-*trans* in acetone
S2 (120 ± 10 fs)
hot S1/ICT (610 ± 10 fs)
S1/ICT (15.68 ± 0.01 ps)
13'-*cis* in acetone
S2 (120 ± 10 fs)
hot S1/ICT (630 ± 10 fs)
S1/ICT (17.18 ± 0.01 ps)
7-*cis* in acetone
S2 (120 ± 10 fs)
hot S1/ICT (640 ± 10 fs)
S1/ICT (25.76 ± 0.02 ps)
15-*cis* in acetone
S2 (130 ± 10 fs)
hot S1/ICT (1.25 ± 0.01 ps)
S1/ICT (12.87 ± 0.01 ps)
9,13'-*cis* in acetone
S2 (170 ± 10 fs)
hot S1/ICT (780 ± 10 fs)
S1/ICT (19.23 ± 0.02 ps)
13-*cis* in acetone
S2 (80 ± 10 fs)
hot S1/ICT (730 ± 10 fs)
S1/ICT (10.81 ± 0.01 ps)
9,13-*cis* in acetone
S2 (60 ± 10 fs)
hot S1/ICT (570 ± 10 fs)
S1/ICT (10.69 ± 0.01 ps)
9-*cis* in acetone
S2 (120 ± 10 fs)
hot S1/ICT (610 ± 40 fs)
S1/ICT (14.53 ± 0.01 ps)
13,13'-*cis* in acetone
S2 (150 ± 10 fs)
hot S1/ICT (450 ± 0.01 fs)
S1/ICT (13.41 ± 0.01 ps)

(b)

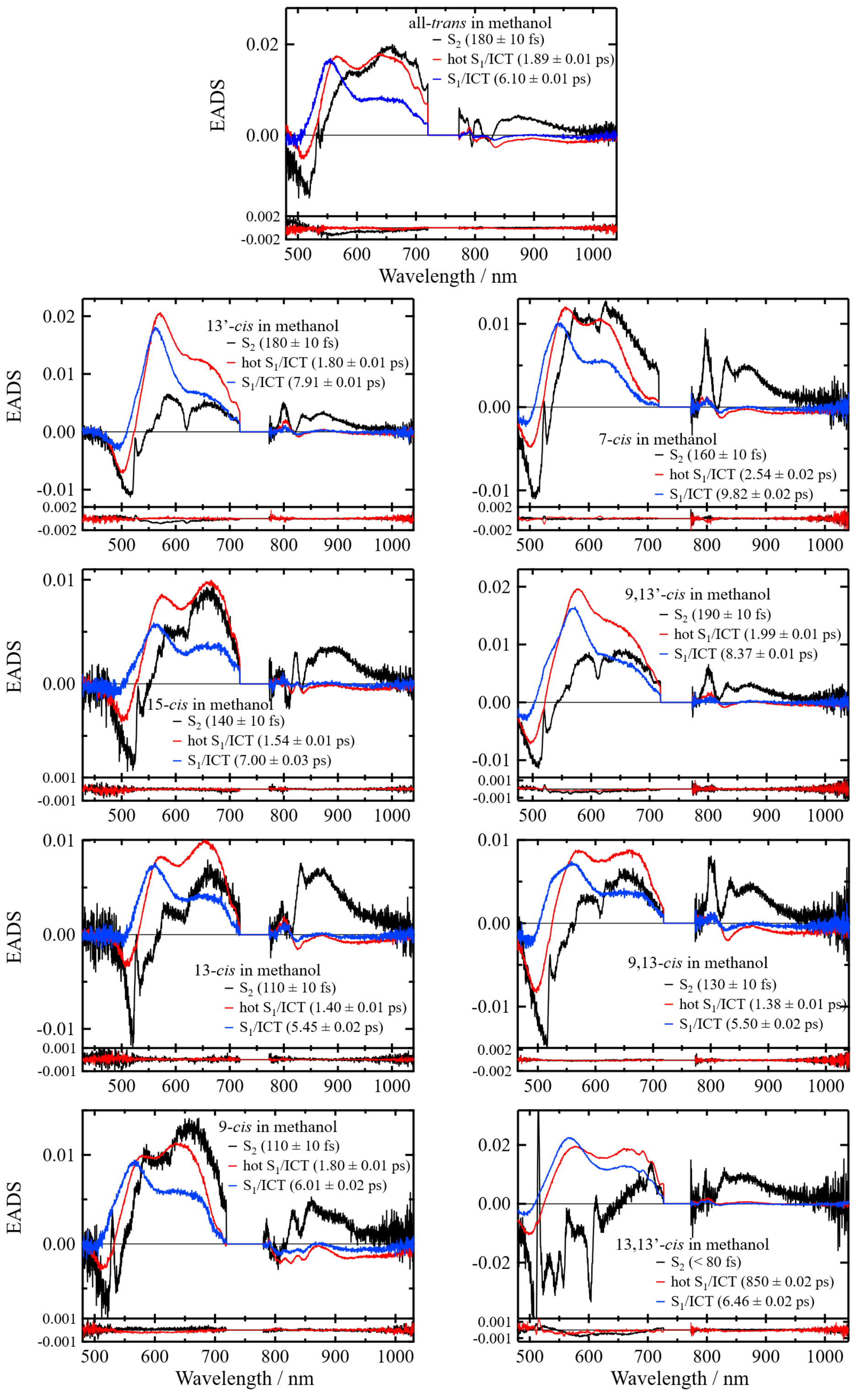

(c)

Figure S4. (a). Evolution associated difference spectra (EADS) of the all-*trans*, 13′-*cis*, 15-*cis*, 13-*cis*, 9-*cis*, 7-*cis*, 9,13′-*cis*, 9,13-*cis*, and 13,13′-*cis* isomers of β-apo-8′-carotenal in *n*-hexane. At the bottom of each panel, the first (black line) and second (red line) right singular vectors of the residual matrix are shown. **(b).** Evolution associated difference spectra (EADS) of the all-*trans*, 13′-*cis*, 15-*cis*, 13-*cis*, 9-*cis*, 7-*cis*, 9,13′-*cis*, 9,13-*cis*, and 13,13′-*cis* isomers of β-apo-8′-carotenal in acetone. At the bottom of each panel,

the first (black line) and second (red line) right singular vectors of the residual matrix are shown. **(c).** Evolution associated difference spectra (EADS) of the all-*trans*, 13′-*cis*, 15-*cis*, 13-*cis*, 9-*cis*, 7-*cis*, 9,13′-*cis*, 9,13-*cis*, and 13,13′-*cis* isomers of β-apo-8′-carotenal in methanol. At the bottom of each panel, the first (black line) and second (red line) right singular vectors of the residual matrix are shown.

5. Estimation of the S1 state energy of the *cis*-isomers of β-apo-8′-carotenal.

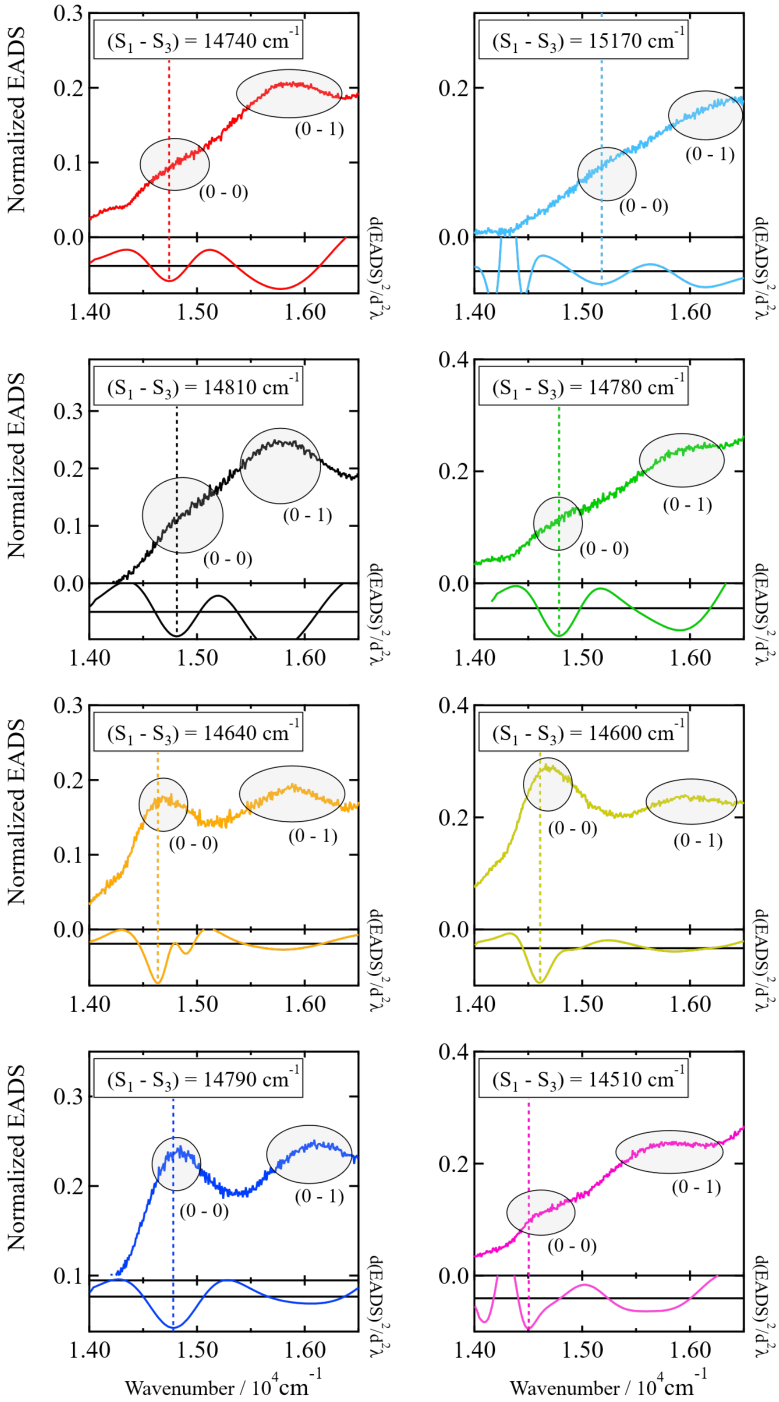

Figure S5. The lower energy-side spectra of the EADS of the S1 species of the isomers of β-apo-8′-carotenal displayed in energy scale, and the results of waveform analysis.

The second derivative waveforms of the EADS were obtained after fitting EADS with multiple Gaussian profiles. The results of the calculations of the second derivatives of EADS are shown at the bottom of each panel.

As illustrated in Figure S5, the lower energy part in the EADS of the S_1 species structured absorption signals are observed (see the spectra of the S_1 species in 600 – 700 nm spectral region in Figure S5-a). The origin of these absorption bands was assigned to the 0-0 and 0-1 transitions of the $S_1 \rightarrow S_3$ absorption of geometric isomers of xanthophylls in the previous study [1]. We have referred to this idea and tried to determine the S_1 state energies of the isomers of β-apo-8′-carotenal. To this end, the second derivative waveforms of the EADS of the S_1 species in the lower energy side (600 – 700 nm spectral region) were calculated, and the 0-0 and 0-1 transition energies of the $S_1 \rightarrow S_3$ transitions of the isomers were determined. Thus determined $S_1 \rightarrow S_3$ transition energies (0-0 transition energies) are summarized in Table S1 together with the 0-0 transition energies of the $S_0 \rightarrow S_3$ absorption (*cis* peak absorption in steady-state spectra).

Table S1. Experimentally determined S_0 – S_3 and S_1 – S_3 gaps of the isomers of β-apo-8′-carotenal in *n*-hexane at room temperature. The S_0 – S_1 energy gaps of the isomers can be calculated by subtracting the S_1 – S_3 energies from the S_0 – S_3 energies. The S_0 – S_1 energy gap of the all-*trans* isomer was determined by fluorescence spectroscopy in the previous study [2].

Isomer	S_0 – S_3 (cm^{-1})	S_1 – S_3 (cm^{-1})	S_0 – S_1 (cm^{-1})
all-*trans*	-	-	15200
7-*cis*	30210	15170	15040
9-*cis*	30400	14790	15610
13-*cis*	30120	14640	15480
15-*cis*	30210	14810	15400
13′-*cis*	30120	14740	15380
13,13′-*cis*	29940	14510	15430
9,13-*cis*	30300	14600	15700
9,13′-*cis*	30210	14780	15430

6. The purities of the *cis* isomers of β-apo-8′-carotenal before and after the femtosecond time-resolved absorption measurements as determined by the HPLC analyses.

Figure S6 shows the results of the HPLC analyses of the *cis* isomers of β-apo-8′-carotenal before and after the femtosecond time-resolved absorption measurements. All the *cis* isomers inevitably show isomerization to all-*trans* during the measurements, although the isomerization starting from the all-*trans* isomer was negligible. However, the extent of isomerization starting from the *cis* isomers was always less than 4% except for the 9,13-*cis* isomer (see Table S2).

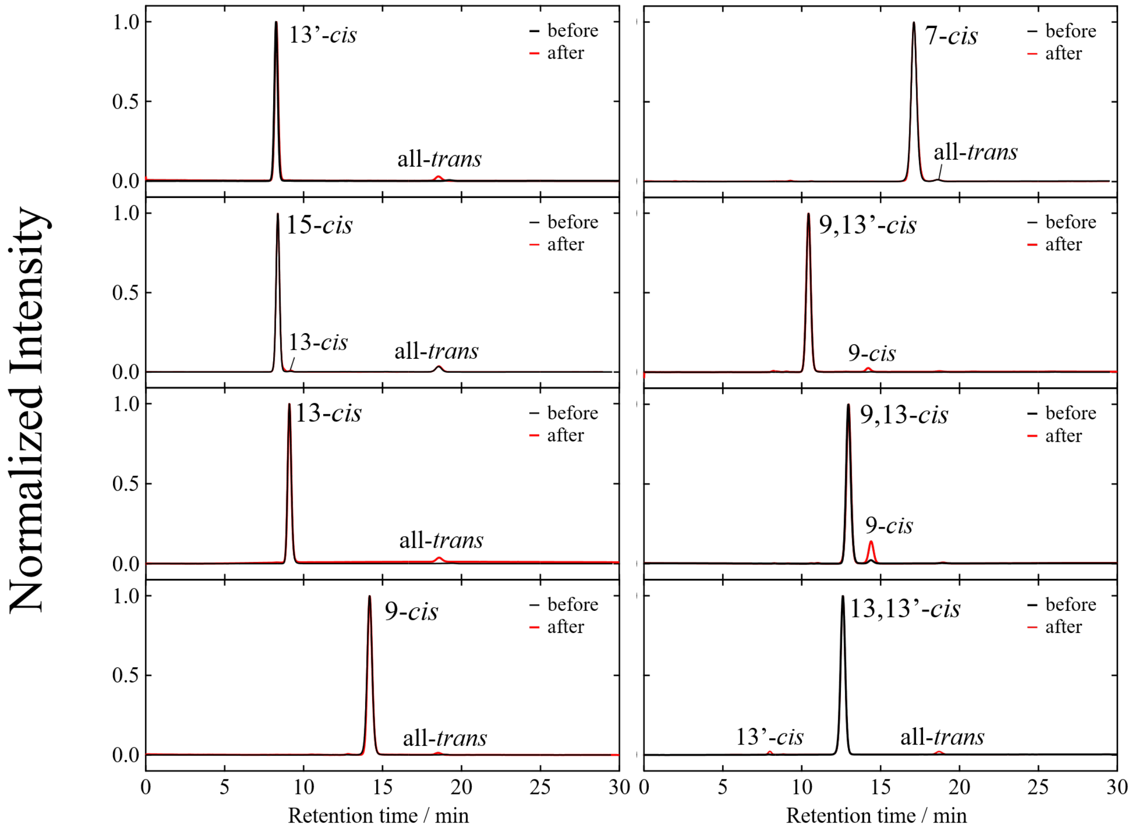

Figure S6. The HPLC profiles of the isomers of β-apo-8′-carotenal before (black) and after (red) the femtosecond time-resolved absorption measurements. Column: Inertsil SIL-100A 4.6×250 mm, 5 μm. Eluent: diethyl ether/*n*-hexane 4/96 v/v. Flow rate: 2.0 mL/min. Detection wavelength: 450 nm.

Table S2. The purity of the isomers of β-apo-8′-carotenal before and after the femtosecond time-resolved absorption measurements.

Isomer	before (%)	after (%)
13′-*cis*	99	96
15-*cis*	95	93
13-*cis*	100	96
9-*cis*	100	99
7-*cis*	99	99
9,13′-*cis*	100	98
9,13-*cis*	98	87
13,13′-*cis*	100	97

References

1. Niedzwiedzki, D.M.; Enriquez, M.M.; Lafountain, A.M.; Frank, H.A. Ultrafast time-resolved absorption spectroscopy of geometric isomers of xanthophylls. *Chem. Phys.* **2010,** *373*, 80-89, doi:10.1016/j.chemphys.2010.01.019.
2. Ehlers, F.; Wild, D.A.; Lenzer, T.; Oum, K. Investigation of the S_1/ICT → S_0 internal conversion lifetime of 4'-apo-β-caroten-4'-al and 8'-apo-β-caroten-8'-al: dependence on conjugation length and solvent polarity. *J. Phys. Chem. A* **2007,** *111*, 2257-2265, doi:10.1021/jp0676888.
